# A study on table tennis landing point detection algorithm based on spatial domain information

**DOI:** 10.1038/s41598-023-42966-6

**Published:** 2023-11-24

**Authors:** Tao Ning, Changcheng Wang, Meng Fu, Xiaodong Duan

**Affiliations:** 1 State Ethnic Affairs Commission Key Laboratory of Big Data Applied Technology, Institute of Computer Science, Dalian Minzu University, Dalian, 116650 China; 2 School of computer Science and Engineering, Dalian Minzu University, Dalian, China

**Keywords:** Engineering, Mathematics and computing

## Abstract

To address the limitations of computer vision-assisted table tennis ball detection, which heavily relies on vision acquisition equipment and exhibits slow processing speed, we propose a real-time calculation method for determining the landing point of table tennis balls. This novel approach is based on spatial domain information and reduces the dependency on vision acquisition equipment. This method incorporates several steps: employing dynamic color thresholding to determine the centroid coordinates of all objects in the video frames, utilizing target area thresholding and spatial Euclidean distance to eliminate interference balls and noise, optimizing the total number of video frames through keyframe extraction to reduce the number of operations for object recognition and landing point detection, and employing the four-frame difference slope method and polygonal area determination to detect the landing point and area of the target object, thereby obtaining precise coordinates and their corresponding areas. Experimental results on the above method on the Jetson Nano development board show that the dynamic color thresholding method achieves a detection speed of 45.3 fps. The keyframe extraction method correctly identifies the landing point frames with an accuracy rate exceeding 93.3%. In terms of drop point detection, the proposed method achieves 78.5% overall accuracy in detecting table tennis ball drop points while ensuring real-time detection. These experiments validate that the proposed method has the ability to detect table tennis ball drop points in real time and accurately in low frame rate vision acquisition devices and real environments.

## Introduction

Detection and tracking of fast small objects play an increasingly important role in the field of sports. In table tennis, badminton, tennis, and other competitions, video-based super slow-motion playback is often used to capture ball landing information as a basis for refereeing. In this paper, we develop algorithms to further assist in the analysis of fast small object landing point detection in sports video sequences by considering the spatial domain and inter-frame relationships of sports videos, and use table tennis as an example for landing point detection analysis. Table tennis balls are small in size, fast in flight, and complex in motion model, and it is difficult to obtain accurate information about the landing point during training, which leads to problems such as difficult review and strong subjective judgment after training, and the detection and analysis of its landing point has become a hot and difficult issue in the field of computer vision. In this regard, researchers at home and abroad have conducted in-depth research and made great progress in this field^1–3^. For example, Zhang^4^ proposed a high-speed stereo vision system using a distributed parallel processing architecture based on a local area network to track table tennis balls, using two smart cameras to photograph and model the table area in 3D, and using dynamic windowing techniques to detect and track the balls, achieving better accuracy in detecting table tennis ball landing points. Myint^5^ proposed adaptive color thresholding and feature-based ball detection under the condition of four low-cost monocular cameras, and achieved a detection tracking accuracy of 91% by reconstructing the 3D trajectory of the ball using the predicted position of the current frame as well as the saved position of the previous frames. Ji^6^ used the LGP + adaboost algorithm to match image areas, and applied the segmentation method based on the eye-movement model and tracking method of a area of interest prediction to table tennis recognition to improve the efficiency and accuracy of table tennis recognition. Gonzalez^7^ determined the table tennis ball coordinates by the consistency of multiple cameras reporting the table tennis ball position, and the detection accuracy and robustness improved as the number of cameras increased. Zhang^8^ combined the color representation properties of both RGB and HSV color models to extract the table tennis ball area and center the table tennis ball using the center-of-mass method, enabling the table tennis ball robot to quickly and precisely locate the table tennis ball in complex environments. Other researchers have used ViBe algorithm^9–11^, optical flow method^12^ and inter-frame difference method^13^ for small object detection. In recent years, neural network methods have taken the detection and tracking of table tennis balls to a new level. Reno^14^ determines whether the image contains tennis balls by classifying small pieces of the input image, which has a high detection accuracy. Yang^15^ used a 3D neural network to fuse the table tennis ball feature information obtained from different channels to identify table tennis balls and table tennis ball landing points using fully connected layers and this method has good results in table tennis ball recognition. Calandre^16^ used a single camera to estimate the size of the ball to obtain the distance from the ball to the camera and used a 2D CNN network for 3D trajectory analysis of the ball, which performed well on the dataset. Kulkarn^17^ used the temporal convolutional network to extract the temporal boundaries of the ball using ball trajectory data to effectively detect serves and misses. Voeikov^18^ proposed a real-time multitasking network architecture based on HD high frame rate cameras to capture table tennis ball landing frames and perform landing table tennis ball recognition with an accuracy of 97% in the single ball case. Komorowski^19^ made the network more suitable for the detection of small targets by improving the single channel deep neural network, which has good performance in the detection of soccer balls. Kamble^20^ proposed the use of a probabilistic bounding box overlap technique to determine the trajectory of a steadily moving ball, with a tracking accuracy of 87.45%. Tan^21^ proposes to design the prior frame of YOLOv4 with K-means clustering, trim the network branches and compress the convolutional layers for table tennis ball size, and use the fast NMS algorithm to accelerate the prediction process and improve the computational speed of the model. Zhao^22^ proposes a table tennis table detection method based on the Temporal Feature Multiplexing Network (TFMN) and Kalman filter, which is capable of achieving high accuracy and real-time detection of table tennis tables.

The above detection algorithms require multi-vision cameras, high frame rate cameras, or the use of neural network algorithms, which are highly dependent on vision acquisition equipment, slow processing speed, and require large amounts of data labeling, making it quite challenging to implement on the ground. To address this problem, this paper uses the target area threshold exclusion method and the spatial Euclidean distance method to exclude noise and interference balls to improve the table tennis ball landing site detection accuracy. The keyframe extraction method is proposed to reduce the number of operations. The four-frame differential slope method is proposed to detect table tennis ball landing outs and reduce the reliance on video capture devices.

This paper is organized as follows: Sect. “Table tennis landing point detection” introduces the methods related to table tennis ball landing point detection. Section “Experimental results and analysis” compares and analyzes the experimental results to verify the effectiveness of the proposed algorithm. Section “Conclusions and future work” summarizes the advantages and disadvantages of the algorithm in this paper and provides an outlook on future work.

## Table tennis landing point detection

### Interference balls and noise exclusion method

**Figure 1:**
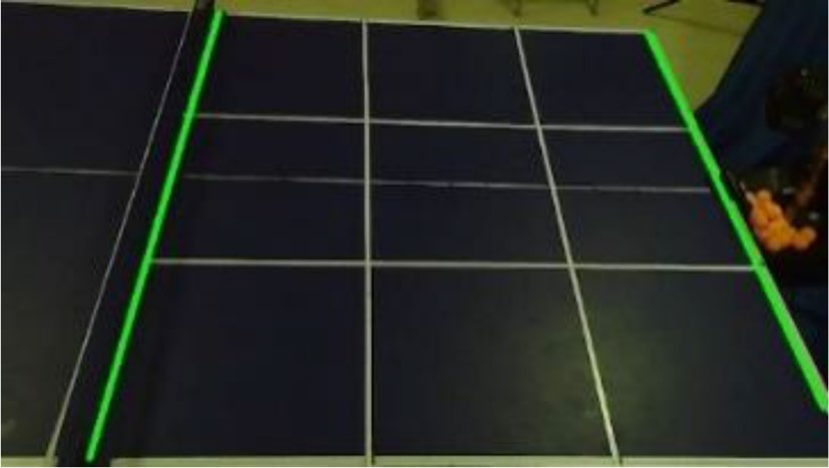
A frame in the video.

The algorithm was tested using a video recorded by a monocular camera at a fixed angle and position under natural lighting conditions. An example frame from the video is shown below, as depicted in Fig. 1.

**Figure 2:**
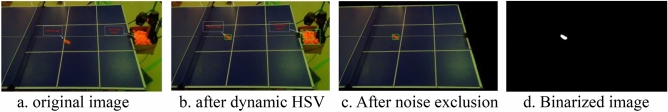
HSV processed effect image.

The color distinction between the table tennis ball and the table in the actual scene is clearly discernible, and the color gamut method is employed for the extraction of the table tennis ball. The color gamut method is a detection algorithm that operates based on the HSV color model. By converting the RGB representation of the picture into H (hue), S (saturation), and V (color brightness) representations, the algorithm allows for precise color analysis. Furthermore, the camera's contrast is adjusted to enhance the quality of the captured images. By defining a specific color threshold interval for the table tennis ball, the algorithm identifies the pixel points falling within the HSV threshold interval as foreground pixels, while considering the remaining pixels as background pixels. This process facilitates the effective extraction of the table tennis ball from the video frame^23^. To address the issue of potential errors arising from the dynamic illumination conditions experienced during each round, a dynamic color threshold method is proposed. This method offers a solution by dynamically adjusting the given HSV threshold according to the specific round being played. To ensure accurate detection of table tennis balls under varying lighting conditions, the proposed method randomly selects 40 table tennis ball pixels located at the tee basket in the first frame of the round. The average HSV values of these selected pixels are then computed and utilized as the color threshold for table tennis balls in that particular round. This approach guarantees the rapid and accurate detection of table tennis balls even in the presence of dynamic illumination conditions. The effect image after HSV processing is shown in Fig. 2.

**Figure 3:**
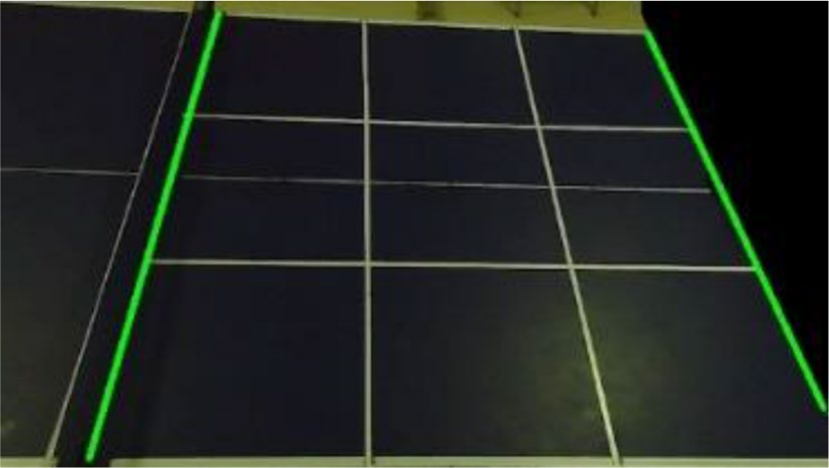
After area shielding.

The area of the table in the video frame constitutes more than 95% of the total area of the frame. To mitigate the risk of false detection, the area of the frame where noise may be present, excluding the table, is masked. The comparison before and after masking is depicted in Fig. 3. Assuming that the contour area of the table tennis ball has been measured in multiple experiments and falls within the interval $[T_{1} ,T_{2} ]$, any objects unrelated to table tennis whose contour areas lie outside this interval are discarded. Let $A_{i}$ represent the set of contour coordinates of all objects extracted from the $i$-th frame after noise reduction. The mathematical model associated with this process is illustrated in Eq. (1).then

$$if\quad T_{1} < a_{n}^{i} < T_{2}$$

1 $$A_{i} = A_{i} \cup \{ C_{n}^{i} (x,y)\}$$

The contour area of the $n$-th object extracted from the $i$-th frame is denoted as $a_{n}^{i}$, and $C_{n}^{i} (x,y)$ represents the set of contour coordinates of the $n$-th object. In the first frame of the round, a fixed value of (655,220) ((655,220) denotes the coordinates of the outlet position of the tee, serving as the initial value of the table tennis ball coordinates before the detection begins. This facilitates the computation of the table tennis ball's position in frame 1 utilizing the Euclidean distance.)is designated as the center of mass for the table tennis ball within the shielded area. Through the combined analysis of the spatiotemporal characteristics of the table tennis balls across consecutive frames, it is observed that the contour coordinates of the table tennis ball in frame $i$ are closest to the center of mass coordinates in frame $(i - 1)$. This holds true even in the presence of minimal noise that cannot be eliminated solely based on the contour area. Let $dist((x_{o}^{i - 1} ,y_{o}^{i - 1} ),(x_{o,n}^{i} ,y_{o,n}^{i} ))$ represent the Euclidean distance between the coordinates $(x_{o}^{i - 1} ,y_{o}^{i - 1} )$ and $({x}_{o,n}^{i},{y}_{o,n}^{i})$, as described by the following Eq. (2).

2 $$dist(({x}_{o}^{i-1},{y}_{o}^{i-1}),({x}_{0,n}^{i},{y}_{0,n}^{i}))=\sqrt{({x}_{o}^{i-1},{x}_{o,n}^{i}{)}^{2}+({y}_{o}^{i-1},{y}_{o,n}^{i}{)}^{2}}$$

$(x_{o}^{i - 1} ,y_{o}^{i - 1} )$ denotes the center-of-mass coordinates of the effective table tennis ball in the $(i-1)$-th video frame. $({x}_{0,n}^{i},{y}_{0,n}^{i})$ denotes the nearest coordinate in $C_{n}^{i} (x,y)$ to $(x_{o}^{i - 1} ,y_{o}^{i - 1} )$.

Remove the interfering table tennis balls by calculating the Euclidean distance between the center-of-mass coordinates of the table tennis balls in frame $(i - 1)$-th image and the contours of all objects in the set $A_{i}$. Suppose $D(x_{o}^{i} ,y_{o}^{i} )$ denotes the set of Euclidean distances of all contours in the coordinates $(x_{o}^{i - 1} ,y_{o}^{i - 1} )$ and $A_{i}$, as shown in Eq. (3).

3 $$D({x}_{o}^{i-1},{y}_{o}^{i-1})=\{dis{t}_{1},dis{t}_{2},dis{t}_{3},\cdots dis{t}_{n-1},dis{t}_{n}\}$$

**Figure 4:**
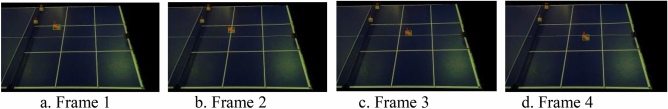
Effective ball table tennis effect illustration(The balls to be excluded are also labeled in the figure, and each number labeled on the top of the rectangular box represents each ball, and the numbers are incremented so that the maximum value represents a valid ball.)

The contour $C_{n}^{i} (x,y)$ corresponding to the minimum dist value within $D(x_{o}^{i} ,y_{o}^{i} )$ is the contour of the valid table tennis balls in the $i$-th frame image. The effective ball is shown in Fig. 4.

### Keyframe extraction method

**Figure 5:**
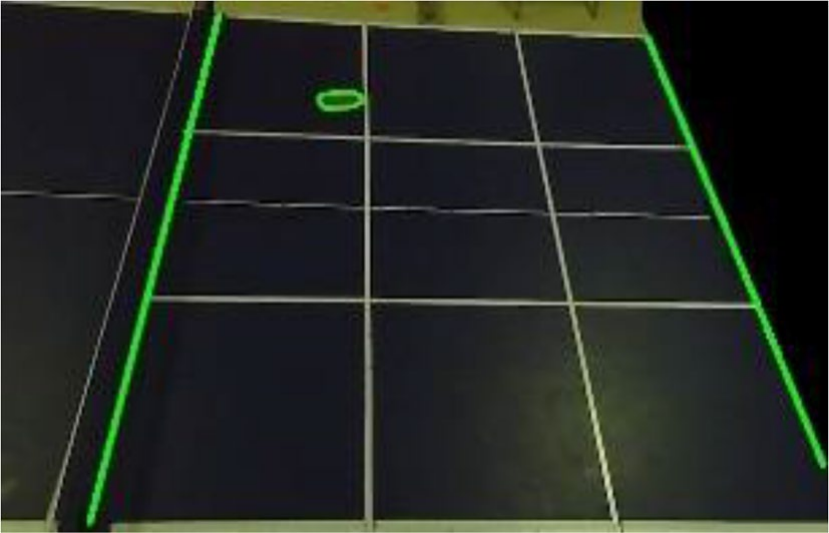
Table tennis shape diagram.

Before extracting keyframes, the center-of-mass coordinates of the obtained valid table tennis ball outline are calculated to determine the ball’s position. When captured using a 60fps camera, a moving table tennis ball produces a long trailing shadow and has a shape resembling a long strip with two short sides protruding. Figure 5 illustrates the captured outline of a table tennis ball, represented by the long green object.

**Figure 6:**
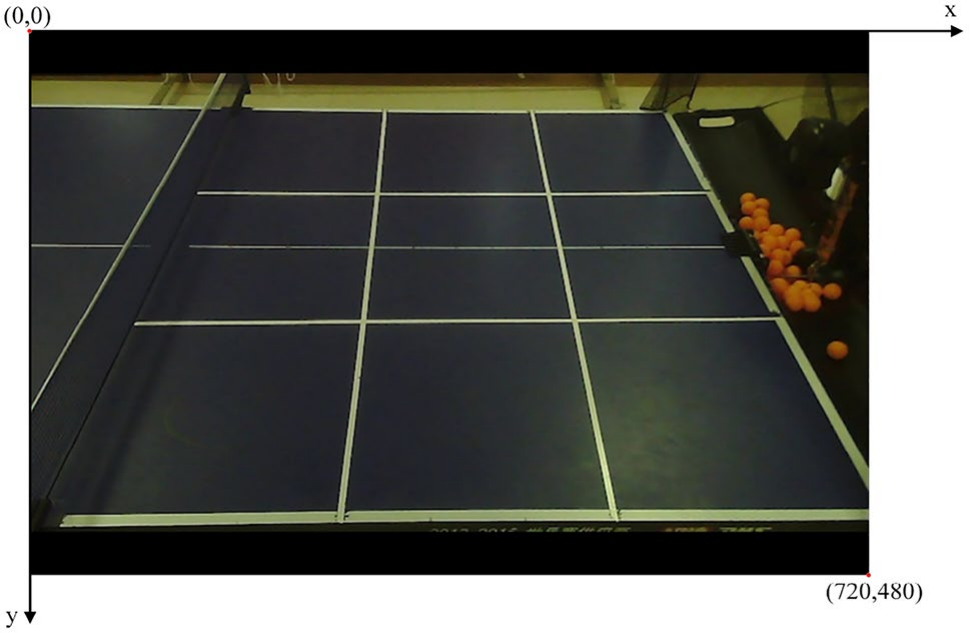
Original picture.

In OpenCV, the upper left corner of the image is used as the coordinate origin, the $x$-axis is the upper horizontal line of the rectangular image, and the $y$-axis is the left vertical line of the rectangular image. As shown in Fig. 6. Suppose the $n$-th coordinate point of the table tennis ball outline in the $i$-th image is $(x_{n}^{i} ,y_{n}^{i} )$, and the set $C_{o}^{i} (x,y)$ of the effective table tennis ball outline coordinate points in the $i$-th image is shown in Eq. (4).

4 $${C}_{o}^{i}(x,y)=\{({x}_{1}^{i},{y}_{1}^{i}),({x}_{2}^{i},{y}_{2}^{i}),({x}_{3}^{i},{y}_{3}^{i}),\cdots ,({x}_{n}^{i},{y}_{n}^{i})\}$$

Suppose the center-of-mass coordinates of the effective table tennis ball in the $i$-th video frame are$(x_{o}^{i} ,y_{o}^{i} )$, and its model is shown in Eq. (5).

5 $$(x_{o}^{i} ,y_{o}^{i} ) = \left( {x_{\min }^{i} + \frac{{x_{\max }^{i} - x_{\min }^{i} }}{2} + \Delta \sigma_{x} ,y_{\min }^{i} + \frac{{y_{\max }^{i} - y_{\min }^{i} }}{2} + \Delta \sigma_{y} } \right)\quad \left( {\Delta \sigma_{x} \le 5,\Delta \sigma_{y} \le 3 pixels} \right)$$

In the set $C_{o}^{i} (x,y)$, $x_{\max }^{i}$ and $x_{\min }^{i}$ denote the maximum and minimum $x$-coordinate values in the set, and $\Delta \sigma_{x}$ and $\Delta \sigma_{y}$ denote the offset values of the table tennis mass center coordinates in the $x$-axis and $y$-axis directions.

**Figure 7:**
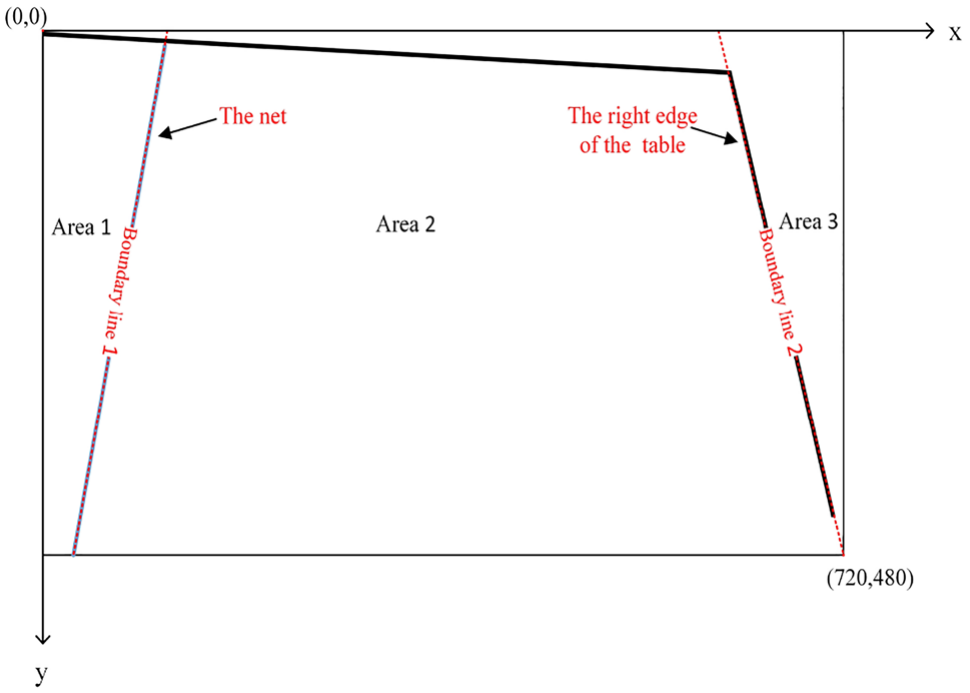
Area division map.

The spatiotemporal characteristics of the obtained table tennis ball center of mass coordinates are analyzed, and the keyframe extraction method is used to retain the frames related to the landing point in the video when the ball moves to the right side of the net, where the landing point may appear, to improve computing efficiency. To calculate the landing point of the table tennis ball on the return side. The video frame image is divided into three areas bounded by boundary line 1 (the net) and boundary line 2 (the right edge of the table) according to the different motion states of the table tennis ball in different areas. The video frame area division is shown in Fig. 7. The tee is located at the center of area 3 shown in Fig. 6, and the tee direction is the negative direction of the $x$-axis. One round starts from the time the tee sends a table tennis ball until the batter hits the ball back to the point where it appears to land in Area 2. The portion of the round from the time the tee sends the table tennis ball until the batter hits the ball back to Area 1 will be the first half of the round, while the subsequent part is used as the second half of this round. Let the equation of the line where boundary line 1 $(l_{1} )$ and boundary line 2 $(l_{2} )$ are located be shown in Eq. (6).

6 $$\left\{ {\begin{array}{*{20}c} {l_{1} = k_{1} \cdot x + b_{1} } \\ {l_{2} = k_{2} \cdot x + b_{2} } \\ \end{array} } \right.$$

Phase 1: The first phase is the serving phase of the tee, which is the first half of a certain round, and 1 frame is taken as a sample frame every 5 frames. The center of mass of the table tennis ball is to the left of $l_{2}$, and the direction of motion of the table tennis ball is in the negative direction of the $x$ coordinate axis. Suppose $F$ denotes the set of sample frames drawn throughout the process, and the relevant expression is shown in Eq. (7).

7 $$if\quad \left\{ {\begin{array}{*{20}c} {f_{i}^{1} \cap \{ f_{i}^{1} ,f_{7}^{1} ,f_{13}^{1} ,f_{6n - 5}^{1} \} \ne \emptyset } \\ {x_{o}^{i} < x_{o}^{i - 1} } \\ {y_{o}^{i} < k_{2} \cdot x_{o}^{i} + b_{2} } \\ \end{array} } \right.$$

$$then\quad F = F \cup \{ f_{i}^{1} \}$$$$f_{i}^{1}$$ denotes the $$i$$-th frame image in the first stage video, and $$f_{6n - 5}^{1}$$ denotes the $$(6n - 5)$$-th frame image in the first stage video $$(n = N^{*} )$$.

Phase 2:The center of mass of the table tennis is to the left of $l_{1}$, and the direction of the table tennis ball is the positive direction of the $x$ coordinate axis, and one frame is taken as the sample frame every three frames.

Phase 3:The table tennis ball center of mass coordinates are within the area enclosed by $l_{1}$ and $l_{2}$, the direction of motion is the positive direction of the $x$ coordinate axis, the table tennis ball landing point appears at this stage, and the video is read frame by frame.

After the table tennis round in the video is run, the set of frames is all the keyframes and sample frames obtained by the keyframe extraction method.

### Four-frame difference slope method

The four-frame differential slope method utilizes the strong spatiotemporal correlation between the center-of-mass coordinates of table tennis balls. It conducts a combined analysis of the center-of-mass coordinates in four consecutive frames from the keyframe set $F$ to determine the landing coordinates of the table tennis ball.

**Figure 8:**
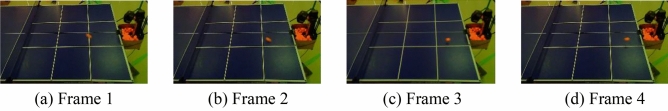
Location map of the 4 frames of table tennis balls adjacent to the landing point.

By observing the trajectory of multiple sets of table tennis balls, we noticed that the direction of motion of the balls changes abruptly after the landing point appears. The slope of the center of mass coordinates of the table tennis ball in the 2 frames before the landing point has the opposite sign to the slope of the center of mass coordinates of the table tennis ball in the 2 frames after the landing point. The coordinates of the intersection of these two slopes represent the landing point of the table tennis ball. The positions of the table tennis ball before and after the landing point are illustrated in Fig. 8. The slope model, which consists of the center-of-mass coordinates of the table tennis ball in 2 frames before and after the landing point, can be described by the following Eq. (8).

8 $$\left\{ {\begin{array}{*{20}c} {k_{i - 2} = \frac{{y_{o}^{i - 2} - y_{o}^{i - 3} }}{{x_{o}^{i - 2} - x_{o}^{i - 3} }}} \\ {k_{i} = \frac{{y_{o}^{i} - y_{o}^{i - 1} }}{{x_{o}^{i} - x_{o}^{i - 1} }}} \\ \end{array} } \right.$$

$(x_{o}^{i - 3} ,y_{o}^{i - 3} ),(x_{o}^{i - 2} ,y_{o}^{i - 2} ),(x_{o}^{i - 1} ,y_{o}^{i - 1} ),(x_{o}^{i} ,y_{o}^{i} )$ denote the coordinates of the center of mass of the table tennis ball in the $(i - 3)$-th, $(i - 2)$-th, $(i - 1)$-th, $i$-th frame, respectively. $k_{i - 2}$ and $k_{i}$ denote the slopes of the lines formed by the table tennis ball center of mass coordinates in the $(i - 3)$-th and $(i - 2)$-th images, the $(i - 1)$-th and $i$-th frame images, respectively. The equation of the line corresponding to the slope and is shown in Eq. (9).

9 $$\left\{ {\begin{array}{*{20}c} {l_{i - 2} = k_{i - 2} \cdot (x - x_{o}^{i - 3} ) + y_{o}^{i - 3} } \\ {l_{i} = k_{i} \cdot (x - x_{o}^{i - 1} ) + y_{o}^{i - 1} } \\ \end{array} } \right.$$

$l_{i - 2}$ denotes the line passing through the table tennis center of mass coordinates $(x_{o}^{i - 3} ,y_{o}^{i - 3} ),(x_{o}^{i - 2} ,y_{o}^{i - 2} )$ with slope $k_{i - 2}$, and $l_{i}$ denotes the line passing through the table tennis center of mass coordinates $(x_{o}^{i - 1} ,y_{o}^{i - 1} ),(x_{o}^{i} ,y_{o}^{i} )$ with slope $k_{i}$ of the line.

The direction of motion of a valid table tennis ball in the data set is the positive direction of the $x$-coordinate axis. When $k_{i - 2} < 0$ and $k_{i} > 0$, the direction of motion of the table tennis ball changes abruptly, and the intersection of the lines $l_{i - 2}$ and $l_{i}$ can be expressed as the landing point, as shown in Eq. (10).

10 $$(O_{x} ,O_{y} ) = (\frac{{y_{o}^{i - 3} - k_{i - 2} \cdot x_{o}^{i - 3} - y_{o}^{i - 1} + k_{i} \cdot x_{o}^{i - 1} }}{{k_{i} - k_{i - 2} }},\frac{{k_{i - 2} \cdot (y_{o}^{i - 1} - k_{i} \cdot x_{o}^{i - 1} ) - k_{i} \cdot (y_{o}^{i - 3} - k_{i - 2} \cdot x_{o}^{i - 3} )}}{{k_{i - 2} - k_{i} }})$$

$(O_{x} ,O_{y} )$ is the coordinates of the landing point of the table tennis ball in this round.

### Table tennis ball landing area determination method

**Figure 9:**
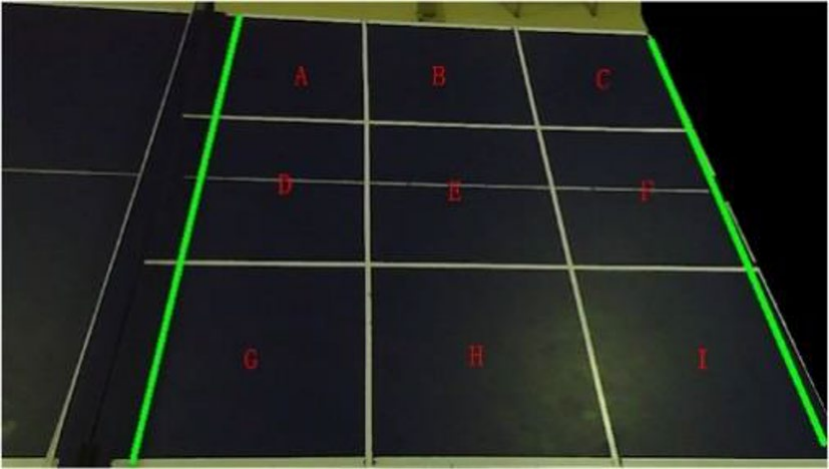
Table area map.

The half of the table tennis table with landing points was divided into 9 areas labeled A-I, as shown in Fig. 9. Due to the camera angle, the quadrilateral formed by the four corner points of the table area is not a rectangle perpendicular to the coordinate axis. To determine the area where the table tennis ball lands, a polygon determination algorithm is proposed.

Taking the I area as an example, after calculating the coordinates of the table tennis ball landing point for a certain round, the vectors formed by the coordinates of each corner point of the I area is multiplied with the vectors formed by $u$ and $v$ respectively, to determine whether this landing point falls within the I area. This is illustrated in Eq. (11).

11 $$\overrightarrow{{I}_{x}^{n},{I}_{x,y}^{n+1}}\cdot \overrightarrow{{I}_{x,y}^{n}{O}_{x,y}}=({I}_{x}^{n}-{I}_{x}^{n+1})\cdot ({I}_{y}^{n}-{O}_{y})-({I}_{n}^{y}-{I}_{y}^{n+1})\cdot ({I}_{x}^{n}-{O}_{x})$$

$I_{x,y}^{n}$ represents the coordinates of the $n$-th corner point of the I area, denoted as $(I_{x}^{n} ,I_{y}^{n} )$. $O_{x,y}$ represents the coordinates of the table tennis ball landing point, denoted as $(O_{x} ,O_{y} )$.$\overrightarrow{{I}_{x}^{n},{I}_{x,y}^{n+1}}$ denotes the vector formed by the coordinates of the $(n + 1)$-th corner point of the I area and the $n$-th corner point in the positive direction, with the clockwise direction considered as the positive direction. $\overrightarrow{{I}_{x,y}^{n}{O}_{x,y}}$ represents the vector formed by the coordinates of the $n$-th corner point of the I area and the coordinates of the table tennis ball landing. If the above operations are performed on the four corner points of the I area, and the four results are the same number, the table tennis ball landing point coordinates $O_{x,y}$ are considered to fall into the I area. Otherwise, the A-H area continues to undergo the same operations until the area that satisfies the conditions is determined.

In summary, the dynamic color gamut method and the interference sphere and noise exclusion method are combined to calculate the coordinates of the effective sphere. The keyframe extraction method is used to reduce the number of operations and increase their speed. The four-frame differential slope method and polygon area determination method are employed to detect the landing positions and locations of the table tennis balls, thereby providing data support for post-match review and refereeing.

## Experimental results and analysis

**Figure 10:**
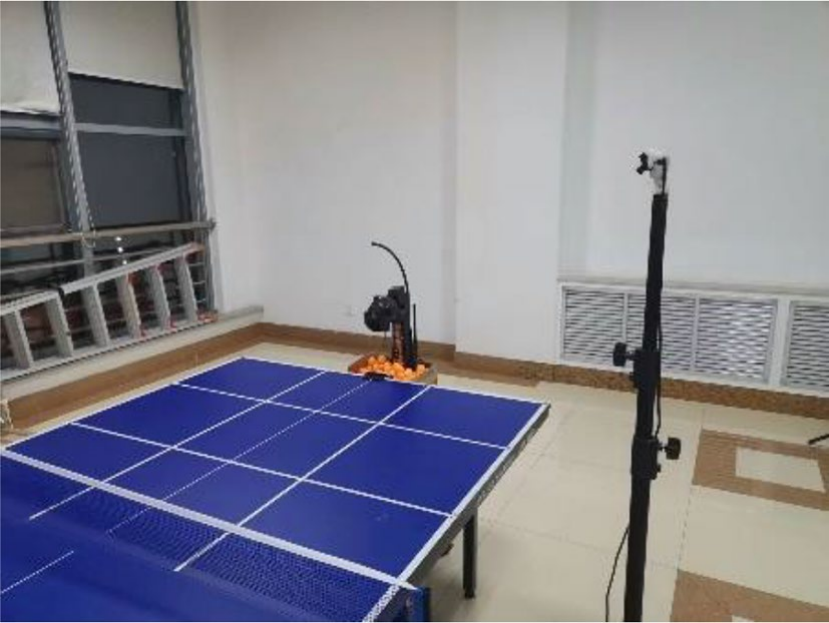
Table tennis table, camera and tee location map.

To verify the effectiveness of the table tennis ball landing point detection and landing area determination methods using human–machine sparring table tennis ball datasets in real scenarios, an experimental platform is utilized. The platform consists of a Jetson Nano equipped with Quadro Tegra X1, ARM CORTEX-A57, and Linux operating system. For video recording, a distortion-free industrial camera with a frame rate of 60fps and a resolution of 720*480 is employed. The camera is positioned 166 cm from the ground and 75 cm from the edge of the table. The table tennis table, camera, and tee are arranged according to the configuration depicted in Fig. 10.

### Analysis of table tennis detection results

The dynamic color thresholding method is employed to extract color objects within the HSV threshold from the global frame image for table tennis detection. This method is known for its speed and robustness against noise. On the other hand, the ViBe algorithm is more suitable for detecting objects with larger deformations and faster motion. In terms of deep learning, YOLO is an algorithm known for its faster detection speed and higher accuracy. To create a labeled dataset for YOLO, 1000 table tennis images were annotated based on the original dataset. In this paper, we compare the traditional ViBe algorithm, its improved version^24,25^, and YOLOv5 as the control detection algorithms. A comparison of the detection speed and accuracy for each algorithm tested in a real scenario is presented in Table 1.

**Table 1:** Comparison of the results of different detection algorithms.

Method	Detection speed(fps)	Detection accuracy (%)
ViBe algorithm	10.4	92.5
Improved ViBe algorithm	18.7	94.1
YOLO v5(tensorRT)	21.1	98.1
Dynamic color gamut method	45.3	93.2

Table 1: The YOLOv5 algorithm has faster detection and excellent overall performance on a high-performance graphics processor, but struggles with inference speed when performing fast small target detection on a lower-performance development board. On the Jetson Nano platform, YOLO v5 achieves a detection speed 24.2 fps slower than the dynamic color thresholding method, while improving detection accuracy by 4.9%, but is unable to achieve real-time detection of small, fast targets. In contrast, the dynamic color thresholding method exhibits significantly higher detection speeds and a better performance balance. The accuracy of the YOLO algorithm is heavily dependent on hardware components such as the camera and graphics processor, making it unsuitable for real-time product development on development boards. The ViBe algorithm demonstrates detection speeds 26.6fps slower than the dynamic color thresholding method. Detection errors are mainly from shadowed foreground objects on the table and image noise in the sphere network. However, with the implementation of noise reduction techniques, the accuracy can be improved to a relatively high level. The improved dynamic color thresholding method excels in both detection speed and accuracy. It provides relatively stable and fast detection results, greatly reduces the video processing time, and enables real-time detection of table tennis balls. The method can be effectively applied to the detection of table tennis balls on low frame rate vision acquisition devices and development boards.

In order to show the relationship between camera frame rate and table tennis ball detection accuracy, the variables other than camera frame rate were controlled to be constant and the results obtained are shown in Table 2.

**Table 2:** Relationship between camera frame rate and detection accuracy.

Method	Camera frame rate(fps)	Detection speed(fps)	Detection accuracy (%)	Camera resolution(pixels)
Dynamic color Gamut method	30fps	45.3	22.5	720 × 480
	60fps	45.3	93.2	720 × 480
	120fps	45.3	93.6	720 × 480

**Figure 11:**
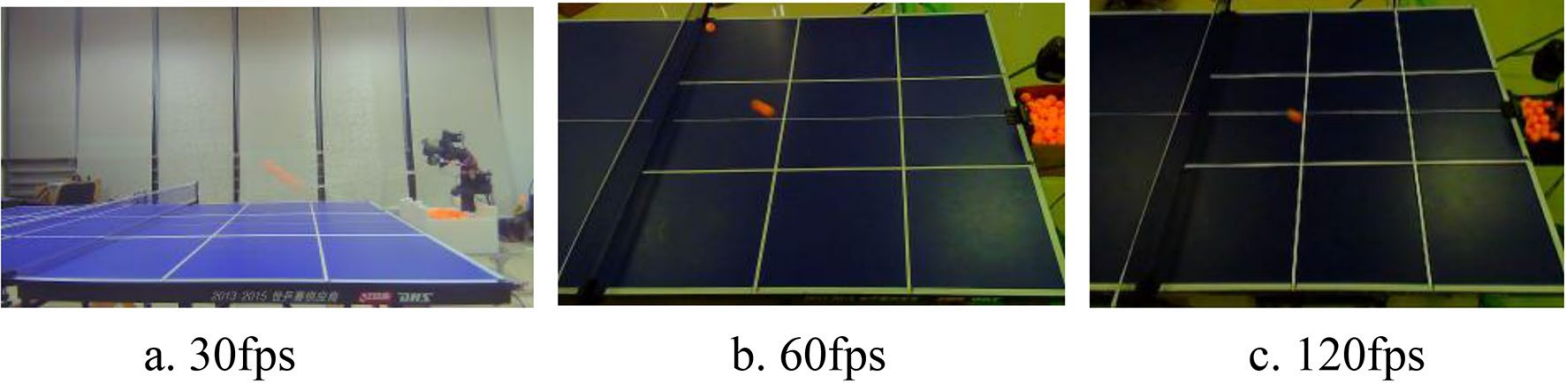
Images captured at different camera resolutions.

Table 2: As can be seen from Fig. 11, when the camera frame rate is 30fps, the drag shadow of the table tennis ball is quite long and the color of the table tennis ball is faded heavily, which makes it difficult to detect the table tennis ball in this case, resulting in the detection accuracy of the table tennis ball of only 22.5%, which is unsuitable to be used for detecting table tennis balls in real situations. When the camera frame rate is increased to 60fps, the table tennis ball drag shadows become substantially shorter and the color of the table tennis ball is basically not faded, and the detection accuracy reaches 93.2%. When the frame rate of the camera reaches 120fps, the detection effect of the table tennis ball is not very different from that of 60fps, but the number of images recorded in the same recording time is increased by one times compared to that of the 60fps camera, which greatly increases the processor's computational capacity and the waiting time. Therefore, the 60fps camera is more in line with the actual situation of table tennis ball detection.

Under the condition of using the dynamic color threshold method, the detection of table tennis balls was performed in the same video. Four frames were randomly selected, and the results before and after adding interference balls and applying the noise exclusion method are shown in Table 3.

**Table 3:** Noise reduction results of interference sphere and noise exclusion method.

	Frame 151	Frame 239	Frame 481	Frame 688
Original image				
No interference ball and noise exclusion method added				
**Add interference ball and noise exclusion method**				

Table 3: The noise observed on the right side of the binarized images of frames 151, 239, 481, and 688, before the inclusion of interference balls and the application of the noise exclusion method, represents interference from table tennis balls in the ball basket of the tee. Additionally, the noise observed on the left side of the binarized image of frame 481 is the misdetected ball net. It can be observed that when using the color gamut method for detection, multiple interfering objects are detected in the image, making it difficult to distinguish the valid table tennis balls. However, with the inclusion of interference balls and the application of the noise exclusion method, interference from noise is avoided, resulting in improved accuracy in table tennis ball landing point detection.

### Analysis of landing point detection results

Table 4: The results presented in the paper demonstrate that the keyframe extraction method processes a significantly lower number of video frames compared to the total number of frames in each video. The number of sample frames and keyframes extracted from each video is less than 32.2% of the total number of frames across all videos. Furthermore, the number of correctly extracted keyframes of the table tennis ball exceeds 93.3%. These findings serve as evidence that, in the majority of cases, the keyframe extraction method accurately captures the frames relevant to the table tennis ball landing.

**Table 4:** Results of the number of video frames before and after keyframe extraction.

Area of the landing point	Correct extraction rate of landing frames (%)	Total number of frames (frames)	Number of keyframes (frames)
B	93.3	3290	912
E	96	3288	896
F	93.3	3420	969
H	96	3261	896
Mixed area 1	93.3	5671	1806
Mixed area 2	93.3	5178	1669

**Figure 12:**
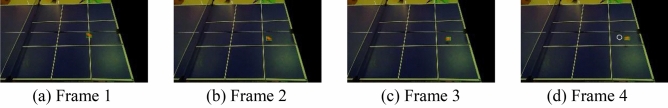
Four-frame difference slope method detection results graph.

Based on the obtained results, a comprehensive analysis was conducted on 350 table tennis balls in the video using three distinct methods: trajectory fitting, TTNet event detection, and four-frame differential slope method. The trajectory fitting method, which is based on the four-frame differential approach^26^, employs the least squares method to accurately model the ball's motion equations before and after contacting the table. The point of intersection between these equations signifies the precise location of the ball's landing point. TTNet, on the other hand, leverages advanced deep learning techniques to detect landing point events by examining a sequence of 9 consecutive frames. Alternatively, the four-frame differential slope method assesses the slope relationship across four consecutive frames of the ball, determining the coordinates of the landing point once specific requirements are met. Subsequent detection is then halted to mitigate computational load. In Fig. 12, the landing point is visually represented by a white circle in subplot (d). The effectiveness of these landing point detection methods is vividly showcased within the visualizations of Fig. 9, while comprehensive accuracy data for all landing points are summarized in Table 5.

**Table 5:** Comparison results of different landing point detection methods.

The method of landing point detection	Accuracy (%)	Speed(fps)
Trajectory fitting method	78.5	24.7
TTNet	97.0	3.7
Four-frame difference slope method	78.5	45.2

Table 5: provides a comparison of the trajectory fitting method, TTNet, and the four-frame differential slope localization method. The results demonstrate that the four-frame differential slope method achieves a significant 20.5fps increase in computational speed compared to the trajectory fitting method. The trajectory fitting method involves segmenting the ball's motion trajectory based on the four-frame differential method, fitting the trajectory, and calculating the intersection. As a result, it requires a substantial amount of computational operations. Furthermore, the trajectory fitting method's performance is affected by the camera's frame rate, leading to difficulties in accurately fitting the trajectory and calculating the landing point when dealing with fast-moving table tennis balls or a limited set of detected coordinates. On the other hand, TTNet is a deep learning algorithm that excels in event detection accuracy. However, it operates at a slower speed, making it more suitable for hardware configurations with higher performance capabilities. In contrast, the four-frame differential slope method mitigates the impact of the vision acquisition device on landing point calculation by discarding subsequent table tennis coordinates once the landing point coordinates are obtained. This approach effectively reduces the overall computational workload. Consequently, the four-frame differential slope method is well-suited for calculating and applying landing point coordinates in table tennis using low frame rate vision acquisition devices and low-computational development boards.

To provide data support for the trainer's training, the program output results are the landing coordinates and landing area of each table tennis ball hit back by the trainer. The landing coordinates and landing area results of the first six balls in the data set obtained using the four-frame differential slope method are shown in Table 6.

**Table 6:** Table tennis coordinates and results of the area to which they belong.

Round number	Coordinates of the calculated landing point	Calculation results for the landing area	Actual area of affiliation
1	(224,337)	D	G
2	(937,326)	F	F
3	(748,277)	E	E
4	(454,526)	H	H
5	(359,219)	B	B
6	(952,626)	I	I

Table 6: The analysis of the landing point area results reveals two main sources of errors in the calculation of the landing point area: (1) When the calculated landing point coordinates fall on the boundary line between two areas, the algorithm assigns the landing point areas based on the dictionary order of the two areas. This can lead to discrepancies between the calculated landing point area results and the actual landing point coordinates, as observed in No. 10. (2) Errors can occur between the calculated landing point area results of a table tennis ball and the actual area it belongs to. The accuracy of the area determination algorithm relies on the precise calculation of the table tennis ball's landing point, as seen in No. 1. In summary, this paper proposes the color field method, which incorporates the four-frame differential slope method for detecting table tennis during landing events. This approach demonstrates significant performance improvements compared to the ViBe algorithm and YOLO algorithm. It is particularly suitable for the development of table tennis landing point detection products intended for low-computational development boards.

## Conclusions and future work

In this paper, we propose a table tennis landing point detection algorithm based on spatial domain information: (1) avoiding false detection by using the target area threshold exclusion method and spatial Euclidean distance method; (2) using the key frame extraction method to discard 67.8% of useless frames in the video to improve the computing efficiency; (3) using the four-frame differential slope method to extract four spatiotemporally connected frames in the video for landing point detection, reducing the amount of computing while weakening the reliance on visual cues. The accuracy of landing point detection reaches 78.5%. For the existing problem that multiple objects cannot be excluded and there are errors in the calculation of the landing area, the neural network algorithm will be used for fast small target object detection in the future, and a combination of global and local methods will be used to improve it, and multiple interfering objects will be excluded according to the movement speed difference and joint spatial intersection ratio method, as well as by adding a monocular camera above the table and using a non-3-dimensional reconstruction method to jointly determine the landing area of the table tennis ball, so as to improve the accuracy of the table tennis ball and its landing point detection.

## Data Availability

The datasets generated and analysed in this study are not publicly available due to project requirements, but are available from the corresponding authors upon reasonable request.
